# Patients with Parkinson’s disease predict a lower incidence of colorectal cancer

**DOI:** 10.1186/s12877-021-02497-z

**Published:** 2021-10-18

**Authors:** Hongsheng Fang, Yunlan Du, Shuting Pan, Ming Zhong, Jiayin Tang

**Affiliations:** 1 Department of gastrointestinal surgery, Renji Hospital, School of Medicine, Shanghai Jiao Tong University, Shanghai, China; 2 Department of Neurology, Renji Hospital, School of Medicine, Shanghai Jiao Tong University, Shanghai, China; 3 Clinical Center for Investigation, Renji Hospital, School of Medicine, Shanghai Jiao Tong University, Shanghai, China

**Keywords:** Colorectal cancer, Parkinson’s disease, Risk, Meta-analysis

## Abstract

**Background:**

Recent theory on the “gut-brain axis” suggests a close relationship between the dysfunction of the gut and the disorders of the brain.

**Methods:**

We performed a systemic literature search followed by a multi-step inclusion selection for all studies on the risk of Colorectal cancer (CRC) in Parkinson’s disease (PD) patients using the following databases: PubMed, EMBASE and WOS. Relative risk (RR) and the 95% confidence intervals (CI) were calculated using either the random-effects model or the fixed-effects meta-analysis model, based on the assessment of heterogeneity.

**Results:**

Seventeen studies involving a total of 375,964 PD patients and 879,307 cancer patients were included. Independent meta-analyses for cohort studies and case-control studies showed that the overall pooled RR of the cohort studies was 0.78 (0.66–0.91), and that of the case-control studies was 0.78 (0.65–0.94), indicating that patients with PD have a significantly decreased risk for CRC. The significant lower risk is present in both the colon and the rectum subgroups classified by tumor location. Moreover, the risk for CRC is significantly lower in America (RR = 0.58), Europe (RR = 0.82) and Asia (RR = 0.83) compared to the control population.

**Conclusion:**

The occurrence of CRC was significantly lower in patients with diagnosis of PD.

**Supplementary Information:**

The online version contains supplementary material available at 10.1186/s12877-021-02497-z.

## Background

Parkinson’s disease (PD) is one of the most common neurodegenerative diseases characterized by motor dysfunction, such as resting tremor, rigidity, hypokinesia and postural instability [1], as well as non-motor symptoms including constipation and depression [2]. The etiology of PD remains unclear, both genetic and environmental factors contribute to it [3]. While the motor symptoms likely resulted from the loss of dopaminergic neurons in the substantia nigra [4], the non-motor symptoms of PD are less well understood. The recent theory on the “gut-brain axis” postulates that the enteric microbiota may influence the cognitive behavior of the brain [5]. On the other hand, the characteristic protein aggregate in the PD brain, α-synuclein, was also found to present in the enteric system [6, 7].

Colorectal cancer (CRC) is the third most common cancer and the second leading cause of cancer death worldwide [8]. CRC ranks the top five cancers in new diagnostic cancers and cancer-related death in China [9], making CRC one of the most serious health problems. CRC develops as the result of the accumulation of genetic and epigenetic alterations [10]. More recently, studies have also suggested that the alteration in the microbiota could generate local and systemic changes to influence oncogenesis [11].

These evidences from both the PD and CRC research fields have suggested a potential interaction between the pathogenic mechanisms of the brain and the gut. Previous epidemiology studies conducted in China showed inconsistent results, indicating the necessity of our research. The goal of the present study is to use the public database to explore the disease risk association between PD and CRC. In the recent 20 years, accumulating epidemiological studies have revealed that patients with PD may be associated with a lower risk of certain cancers [12–14], however, the association between PD and CRC remains controversial [15]. Therefore, we conducted this meta-analysis to provide a quantitative assessment of current epidemiological evidence on CRC in relation to PD and to explore the potential factors affecting the association between the two.

## Methods

### Literature search

Relevant studies from January 2000 to April 2020 were collected from the three major online databases including PubMed, Web of Science and EMBASE by two independent investigators (Hongsheng Fang and Jiayin Tang). We input PARKINSON DISEASE, PARKINSONISM, TUMOR, NEOPLASM, and CANCER as Medical Subject Heading (MeSH) terms and then connected through Boolean operators. We placed no restrictions on the region of residence or the age of the subjects, but we restricted the search to studies including human study participants. Moreover, the relevant reviews and references of articles were also manually screened to identify additional related studies that may supply relevant data. We conducted this meta-analysis according to the recommendations of the Preferred Reporting Items for Systematic Reviews and Meta-analysis (PRISMA) [16].

### Eligibility criteria

Inclusion criteria were defined as follows: (1) Studies are either cohort or case-control studies about the CRC risk of PD patients (Secondary processing articles such as meta-analyses and reviews were excluded.); (2) An estimate of association [e.g. odds ratio (OR), relative risk (RR), hazard ratio (HR) or standardized incidence ratio (SIR) and a 95% confidence interval (CI)] can be collected from the study; (3) The distribution data could be obtained by contacting the author of a relevant report; (4) Studies contain the risk of CRC after the diagnosis of PD (Studies concerning the risk of CRC before the PD diagnosis were excluded.); (5) When duplicated studies were identified, only the most informative study was included.

### Quality assessment and data extraction

The quality of the included articles was scored using the Newcastle-Ottawa scale (NOS) [17] by two investigators (Hongsheng Fang and Jiayin Tang) independently (Table S1), studies with NOS scores > 6 were considered high-quality studies. A third reviewer was recruited when disagreement rises. Data were extracted from eligible studies including author, publication year, type of study design (cohort or case-control), sample size, region, adjustment factors, RR and the 95% CI.

### Statistical analysis

STATA 15.0 (Stata Corporation, College Station, TX, USA) was used to perform data analyses in this study. The pooled relative risk (RR) at the 95% confidence interval (95% CI) was assessed to evaluate the association between PD and CRC. The RR and the 95% CI was calculated using the “inverse variance” method; and the statistical heterogeneity was evaluated using the I^2^ statistics [18]. The fixed-effects meta-analysis model was used when I^2^ is less than 50% and the random-effects meta-analysis model was used when I^2^ is higher than 50% [19]. The RR was used instead of other related measures (such as OR、HR or SIR) because the incidence of PD and CRC are both rare. The effect of publication bias was evaluated by the Begg’s and Egger’s tests [20]. Statistical significance was determined at *P* value less than 0.05.

## Results

### Eligible studies

**Fig. 1:**
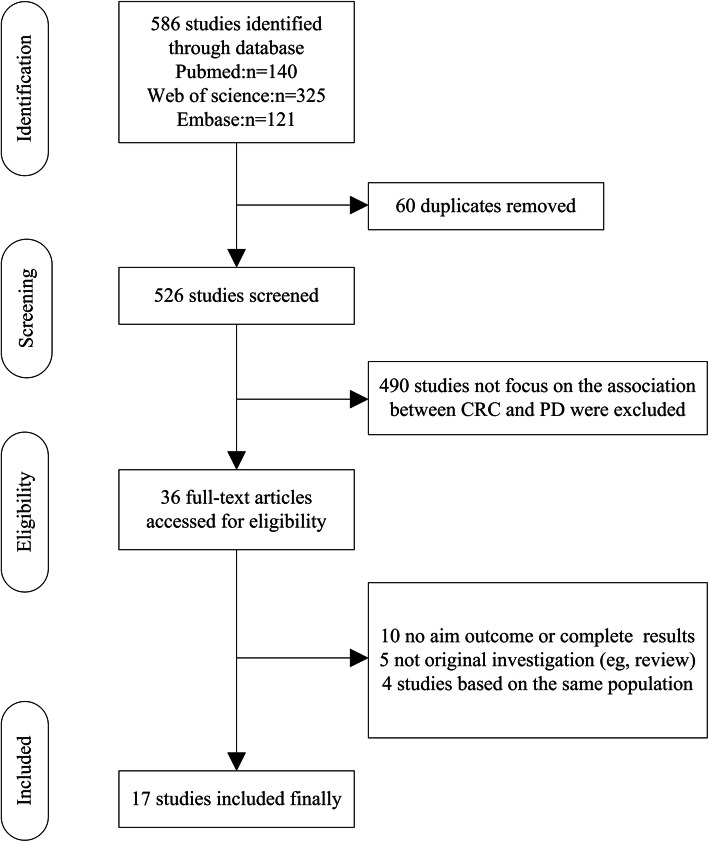
PRISMA flow chart of literature searches and results

The systemic search result and the subsequent eligibility selection workflow is shown in Fig. 1. Our initial search identified a total of 586 potential match in the database. After exclusion of 60 duplicate studies, 526 studies remained. We next excluded 490 studies that fail to provide information on the association between PD and CRC, leaving 36 full-text articles. After a careful review of the remaining 36 studies, 19 studies did not meet our 5 inclusion criteria above (10 studies with no aim outcome or complete results, 5 studies are not original investigation such as review; 4 studies were performed on the same population). Finally, we collected 17 eligible studies for further analyses. All 17 studies were published between January 2000 and April 2020, which included 13 cohort studies (12 retrospective studies and 1 prospective study) and 4 case-control studies. To classify the 17 studies by geographical region, 7 studies were based on the European population [21–27], 6 studies are performed in American population [28–33], while 4 studies are conducted in Asian population [14, 15, 34, 35]. The baseline characteristics of all included studies are shown in Table 1.

**Table 1:** Baseline characteristics of all included studies in the meta-analysis

Author	Year	Country	Study design	N (case)	N (control)	subsite	SQ	adjustment
Guttman [28]	2003	Canada	retrospective cohort	15,304 PD	30,608	colon	6	age, sex
OLsen [21]	2005	Denmark	retrospective cohort	14,088 PD	NR	colon +rectum	6	age, sex
Powers [29]	2005	USA	Case-control	352 PD	484	colorectal	6	age, ethnicity, education, smoking
Driver [30]	2007	USA	prospective cohort	487 PD	487	colorectal	6	age
Fois [22]	2009	UK	retrospective cohort	4355 PD	574,860	colon +rectum	7	age, sex, year of first hospital admission, region
Becker [23]	2010	UK	retrospective cohort	2993 PD	3003	colorectal	6	age, sex, smoking, body mass index,
Lo [31]	2010	USA	retrospective cohort	692 PD	761	colorectal	7	age, sex, ethnicity, education (years), annual income, smoking, alcohol consumption, body mass index
Sun [14]	2011	Taiwan, China	retrospective cohort	4957 PD	19,828	colorectal	6	age, sex
Rugbjerg [24]	2012	Denmark	retrospective cohort	20,343 PD	32,360	colorectal	7	age, sex, calendar year
Ong [25]	2014	UK	retrospective cohort	219,194 PD	9,015,614	colon +rectum	8	age, sex, calendar year, region of residence, quintile of patients
Wirdefeldt [26]	2014	Sweden	retrospective cohort	11,786 PD	58,930	colon +rectum	7	sex, birth year
Lin [15]	2015	Taiwan, China	retrospective cohort	62,023 PD	124,046	colorectal	8	age, sex
Peretz [34]	2016	Israel	retrospective cohort	7125 PD	NR	colon +rectum	7	age, sex, chronological year
Boursi [27]	2016	UK	Case-control	22,093 cancer	85,833	colorectal	6	Obesity, diabetes, smoking, alcohol consumption, NSAIDs use, hormone replacement therapy, screening colonoscopy
Freedman(1) [33]	2016	USA	Case-control	836,947 cancer	142,869	colon +rectum	5	age, sex, selection year
Freedman(2) [32]	2016	Asia	Case-control	20,267 cancer	5558	colon	5	age, sex, selection year
Park [35]	2019	Korea	retrospective cohort	52,009 PD	260,045	colorectal	8	age, sex, hypertension, DM, hyperlipidemia

*Notes: Study quality was judged based on the Newcastle-Ottawa Scale,*

*Abbreviations: N number of studies, NR not reported, SQ score of study quality, RR relative risk, CI confidence intervals*

### Overall association between CRC and PD

**Fig. 2:**
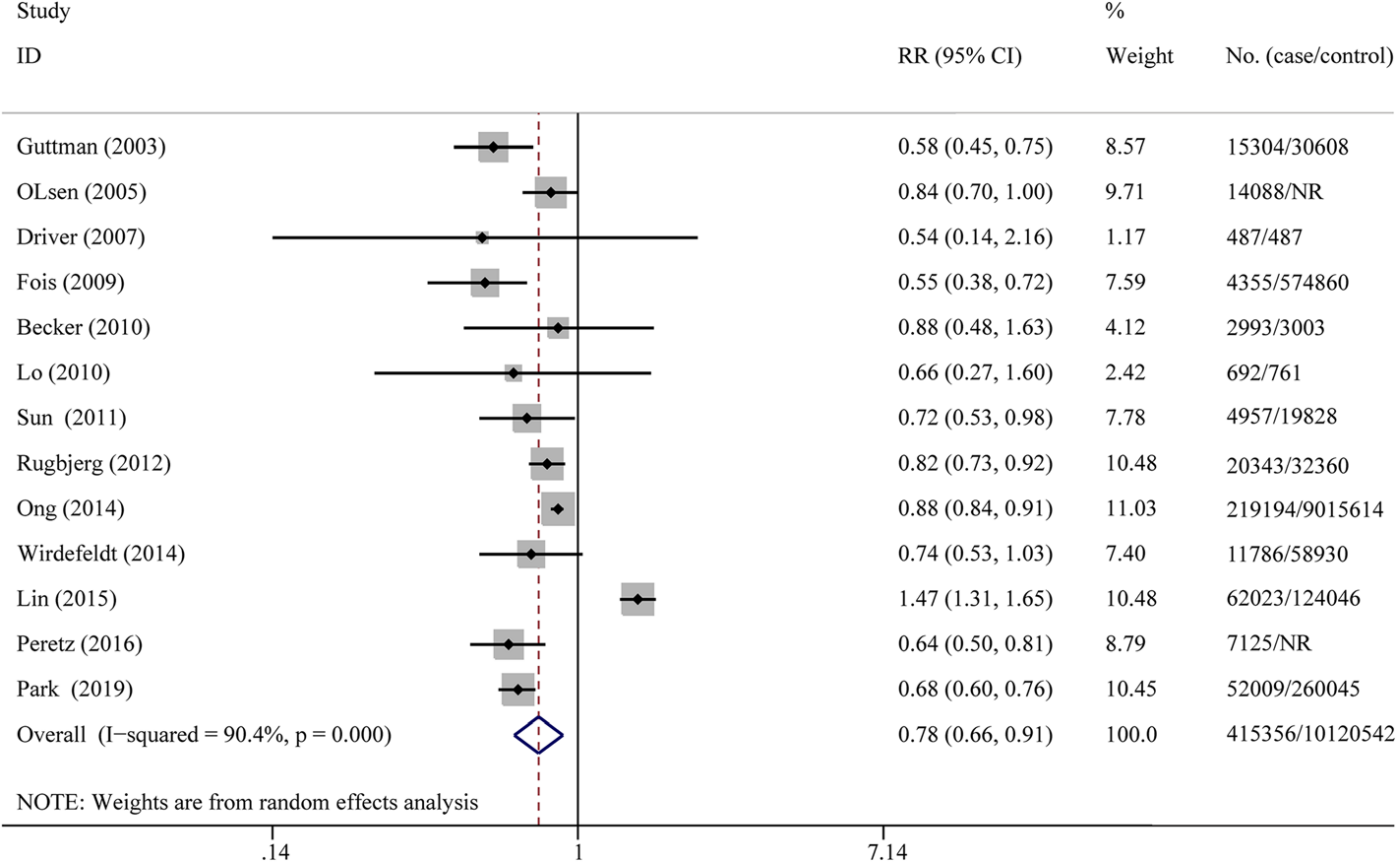
Forest plot of RRs for risk of CRC among PD patients (cohort study)

**Fig. 3:**
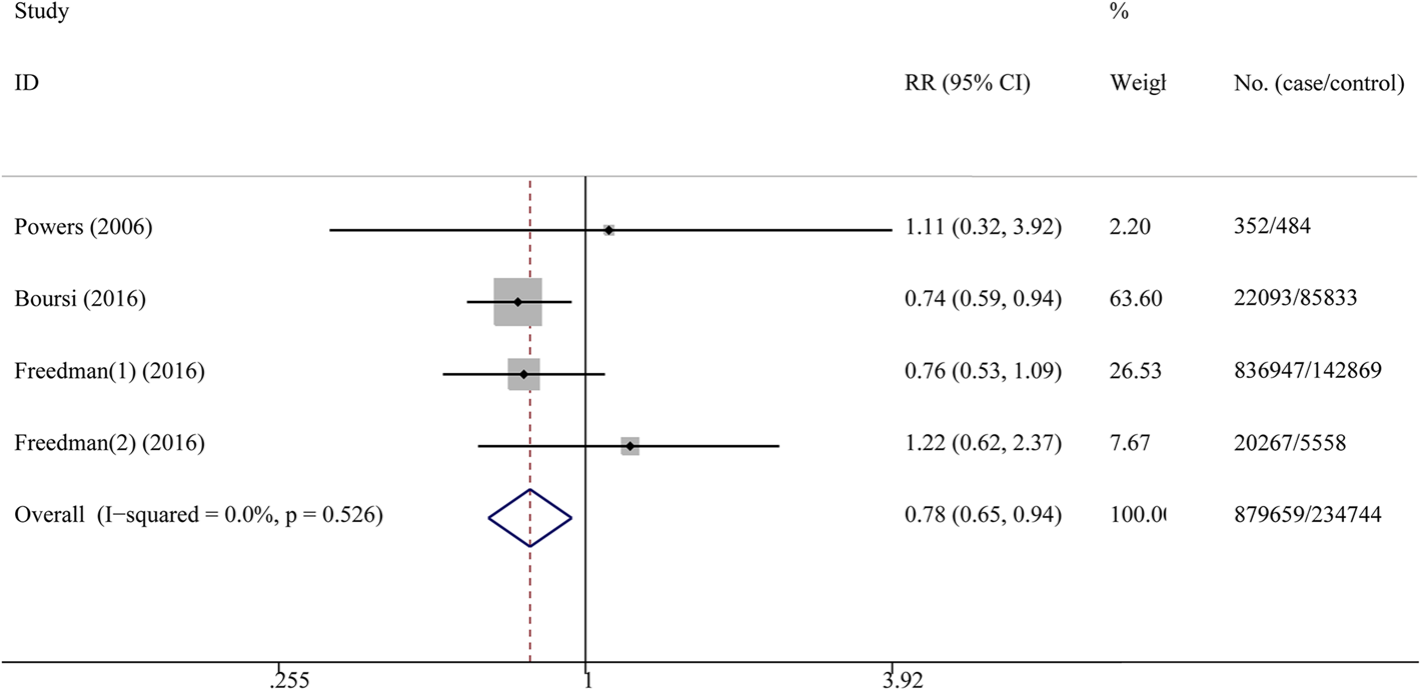
Forest plot of RRs for risk of CRC among PD patients (case-control study)

The pooled RRs of the overall CRC risk in PD patients was 0.78 (95% CI: 0.66–0.91, *p*<0.001, I^2^ = 90.4% = in the cohort studies (Fig. 2) and 0.78 (95%CI: 0.65–0.94, *p*<0.001, I^2^ = 0% = in the case-control studies (Fig. 3), indicating that the PD patients are associated with an overall decreased risk of CRC compared with the control population. However, we observed that the I^2^ is more than 50% in the cohort studies, suggesting high heterogeneity. To understand the source of this unusually high heterogeneity, we examined all studies and found one with the highest OR of 1.47 [15]. By excluding this particular study, we reduced the heterogeneity by nearly 18.3% (heterogeneity I^2^ = 72.1%).

### Results of subgroup analysis

We carried out a series of subgroup analyses based on the tumor location and the region of population, subgroups are selected only from the cohort studies (Table 2). We found that the inverse risk association between PD and CRC persisted even after stratifying the studies by the above factors. Subset analyses of the two study designs showed a consistently decreased cancer risk, with a combined RR of 0.80(*p*<0.001 = for cohort studies and 0.76 (*p*<0.001) for case–control studies. In analyses stratified by tumor location, the combined risk for colon tumor with PD is 0.68 (95% CI, 0.55–0.83, *p*<0.001) (Fig. S1), and that for rectum tumor with PD is 0.89 (95% CI, 0.83–0.95, *p*<0.001) (Fig. S2). To segregate the data by geographical region (Table 2), the average OR was the lowest in data from the American population (OR = 0.58; 95% CI, 0.46–0.74, *p*<0.001) (Fig. S3), followed by data from the European population (OR = 0.82; 95% CI, 0.74–0.9, *p*<0.001) (Fig. S4), in the data obtained from Asia, the OR is 0.83 with 95% CI (0.51–1.34, *p* = 0.442) (Fig. S5). PD patients in these areas showed significantly lower CRC risk compared to the control population. It is worth noting that the heterogeneity (I^2^) in the Asia group would reduce from 97 to 0% if excluding the study with OR of 1.47.

**Table 2:** Subgroup analysis

Categories	N	Pooled RR	95% CI	P value	Heterogeneity	
					I^2^	P′
Study design						
Cohort	13	0.78	0.66–0.91	<0.001	90.4%	<0.001
Case-control	4	0.78	0.65–0.94	<0.001	0%	0.526
Cancer location						
Colon	6	0.68	0.55–0.83	<0.001	86.2%	<0.001
Rectum	5	0.89	0.83–0.95	<0.001	0	0.887
Geographical region						
America	3	0.58	0.46–0.74	<0.001	0	0.957
Europe	6	0.82	0.74–0.90	<0.001	51%	0.070
Asia	4	0.83	0.51–1.34	0.442	97%	<0.001

*Abbreviations: N number of studies, RR relative risk, CI confidence intervals, P′ p value of I*^*2*^ *statistics for heterogeneity*

### Sensitivity analysis

In our meta-analysis, sensitivity analysis was conducted to assess the stability of the results. The persistent inverse association between PD and CRC risk did not change in the sensitivity analysis, which was conducted by omitting one study per iteration. (Fig. S6).

### Publication bias analysis

We used Begg’s and Egger’s tests to evaluate publication bias in this meta-analysis. The funnel plot was almost symmetric (Fig. S7), the Egger test for publication bias (*p* = 0.319) showed no significant evidence for bias in the data, Begg’s test (*p* = 0.951) was also not strongly suggestive of publication bias, thus confirming the absence of obvious publication bias in these studies (Fig. S8).

## Discussion

PD is an age-related neurodegenerative disorder commonly diagnosed at the age of 60 and above. CRC is increasingly common in people over the age of 60. PD and CRC are totally different illnesses and are thought to have different pathogenic mechanisms. For example, PD is characterized by the death of dopaminergic neurons in the substantia nigra, while CRC derives from the inappropriate cell proliferation with a selective growth advantage. Growing evidence have suggested that patients with PD may have a substantially lower incidence of cancer, but the risk association with CRC is less clear.

Prior to our study, there was only one meta-analysis examining the association between PD and CRC. Unfortunately, there were several caveats and weaknesses associated with that study: (1) It was unclear if the results represented the risk of cancer influenced by PD, or vice versa; (2) The data collected in that study was incomplete; (3) It is methodologically wrong to mix the results from the cohort studies and the case-control studies due to the different design of the two studies.

Our study has overcome the aforementioned weaknesses in the previous work and examined specifically the risk of CRC in patients with diagnosis of PD. We analyzed 17 studies involving 375,964 PD patients and 879,307 cancer patients. The pooled results for all populations indicated that PD patients have a decreased risk of CRC in Western population. Subgroup analysis showed that the significant inverse relationship between PD and risk of CRC is not affected by differences in types of study design, tumor location, or different regions of the Western population.

The inverse association between PD and CRC in the Western population is statistically significant in our study. However, the association in the Asian population remains obscure. We found contradictory conclusions in published studies. In our meta-analysis, we identified a modest lower risk of CRC in patients with PD (RR = 0.83) with a high level of heterogeneity (I^2^ = 97%) and the significance is not as clear as data from the American and European groups(*P* = 0.442). We think the reason could be due to the wide variability of the Asian data. Some large-scale studies on the Asian population are unfortunately not completed like the similar studies for the Western population. For the two studies that are completed in Taiwan, China, the results are opposite. The RR of the study completed by Sun [14] in 2011 is 0.72 (95% CI 0.53–0.99). The study consisted of 4957 newly diagnosed PD cases in the cohort and 19,828 non-PD controls during the period of 2000–2005 from the Taiwan National Health Insurance (NHI) Research Database (NHIRD), a nationwide population-based database containing more than 24 million subjects covering 99% of the entire population in Taiwan, China. The other study that completed by Lin [15] in Taiwan, China in 2015 is the main source of heterogeneity, the pooled OR of this study is 1.47 (95% CI, 1.31–1.65), and the cohort study included 133,322 individuals with PD newly diagnosed between 2004 and 2010, also from the NHI database. Freedman [32] et al. adjusted for a surrogate for surveillance (number of physician visits) and found that the odds of cancer in the total population after PD was reduced, suggesting that the medical surveillance contributed to the risks.

The key question that our study begs is the potential mechanism that may account for the negative association between PD and CRC. The level of melatonin [36], dopamine [37], smoking [38] and diabetes [39] have all been proposed to account for such mechanisms. More importantly, patients with PD often have microflora alterations in their feces and colonic mucosa, which may lead to non-motor symptoms such as constipation [40]. Interestingly, recent studies have also pointed to the role of microbiome and their secretion in inducing local and systemic effects on cancer onset and progression [11]. Thus, a detailed analysis on the types of microbiota may provide clues to a negative association between PD and CRC. At the molecular level, dysfunction of the ubiquitin-proteasome system (UPS) leads to an accumulation of intracellular proteins and formation of Lewy bodies containing α-synuclein, which is the characteristic pathological feature of PD [41, 42]. In contrast, the function of UPS is usually up-regulated in CRC [43]. Whether the UPS stands at the crossroads of dysregulation for PD and CRC awaits further studies. Moreover, studies have shown that the PI3K /AKT/mTOR pathways are hyperactive in patients with CRC [44] while the activation of the PI3K/AKT/mTOR pathway may promote the survival of dopaminergic neurons by inhibiting apoptosis, thus preventing PD [45].

Our meta-analysis has provided a most up-to-date picture for the CRC risk in PD patients. As CRC patients often exhibit a much more aggressive disease course than the PD patients, the risk of PD in CRC patients is hard to calculate. Our study, thus, made a strong conclusion for the inverse CRC risk from the Western PD population, while that for the Asian population remains obscure due to large heterogeneity and a small number (4) of available datasets. It is worth noting that publication bias and other forms of bias may still exist, and a more detailed subgroup analysis is incomplete due to the insufficient data from the primary articles. Nonetheless, our study has several strengths, including its comprehensive literature search for the latest data, large number of cases, careful assessment of the quality of evidence, which altogether made the results more reliable compared to earlier studies.

## Conclusion

In conclusion, our research suggests that patients with PD predict a lower risk of CRC. Further studies are warranted to explore the underlying mechanisms of this correlation and to prevention and treatment of both diseases.

## Supplementary Information

**Additional file 1: Fig. S1** Forest plot of subgroup analysis (colon). **Fig. S2** Forest plot of subgroup analysis (rectum). **Fig. S3** Forest plot of subgroup analysis (America). **Fig. S4** Forest plot of subgroup analysis (Europe). **Fig. S5** Forest plot of subgroup analysis (Asia). **Fig. S6** Sensitivity analysis of cohort study. **Fig. S7** Funnel plot of cohort study. **Fig. S8** Egger’s tests of cohort study.

**Additional file 2: Table S1** The quality of the included studies assessed by NOS.

## Data Availability

All data generated or analyzed during this study are included in this published article and its supplementary information files.

## Abbreviations

RR: Relative risks
OR: Odds ratio
HR: Hazard ratio
CI: Confidence intervals
NR: not reported
SQ: score of study quality
N: number of studies
P′: *p* value of I^2^ statistics for heterogeneity
PRISMA: Preferred Reporting items for System Review and Meta-analysis
NOS: Newcastle–Ottawa Scale
