# Supplementary material for: Patients with Parkinson’s disease predict a lower incidence of colorectal cancer
Source: BMC Geriatr. 2021 Oct 18;21:564. doi: 10.1186/s12877-021-02497-z (PMC8522030; doi:10.1186/s12877-021-02497-z)

**Patients with** **Parkinson’s disease predict a lower incidence of colorectal cancer**

Hongsheng Fang, Yunlan Du, Shuting Pan, Ming Zhong, Jiayin Tang


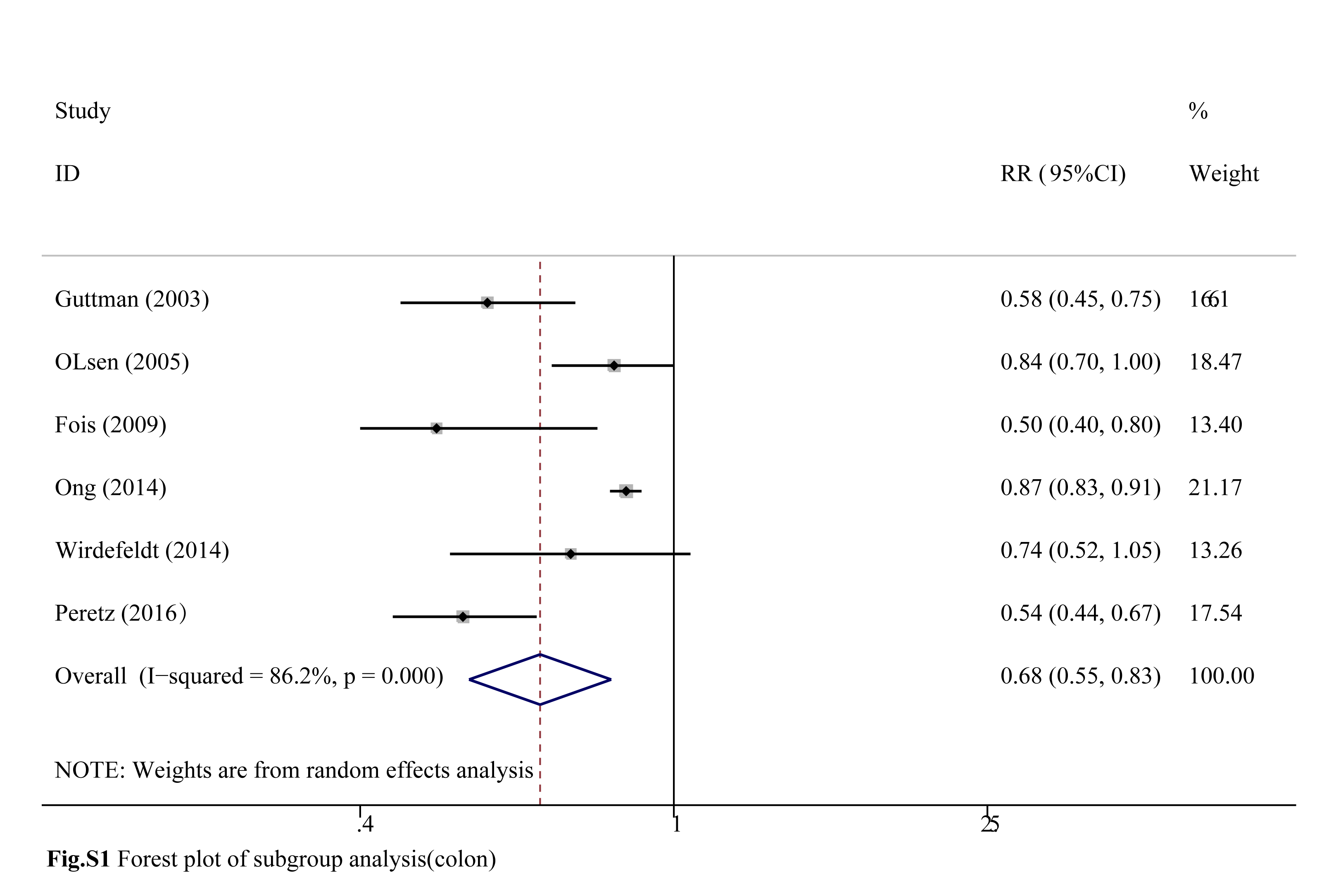


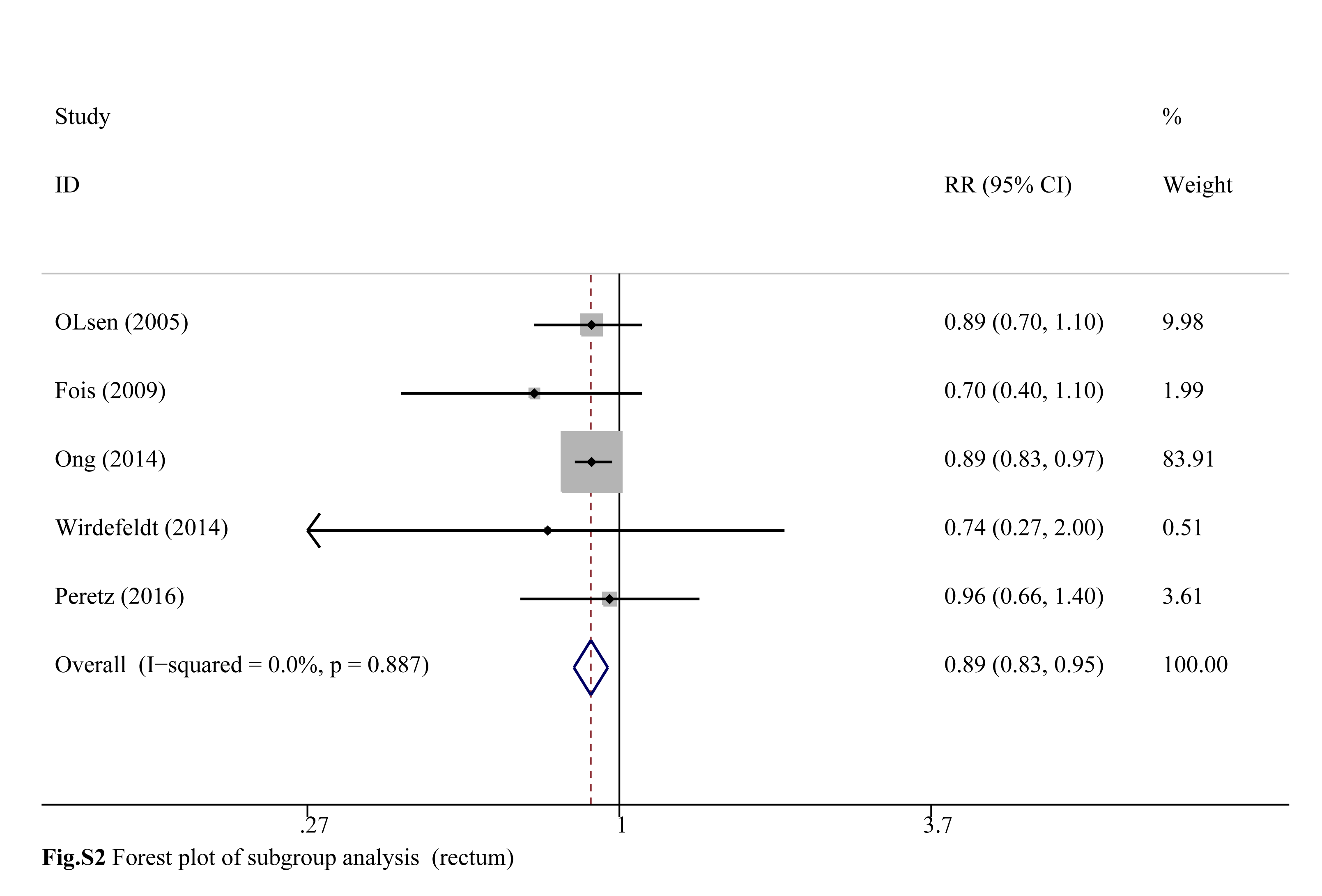


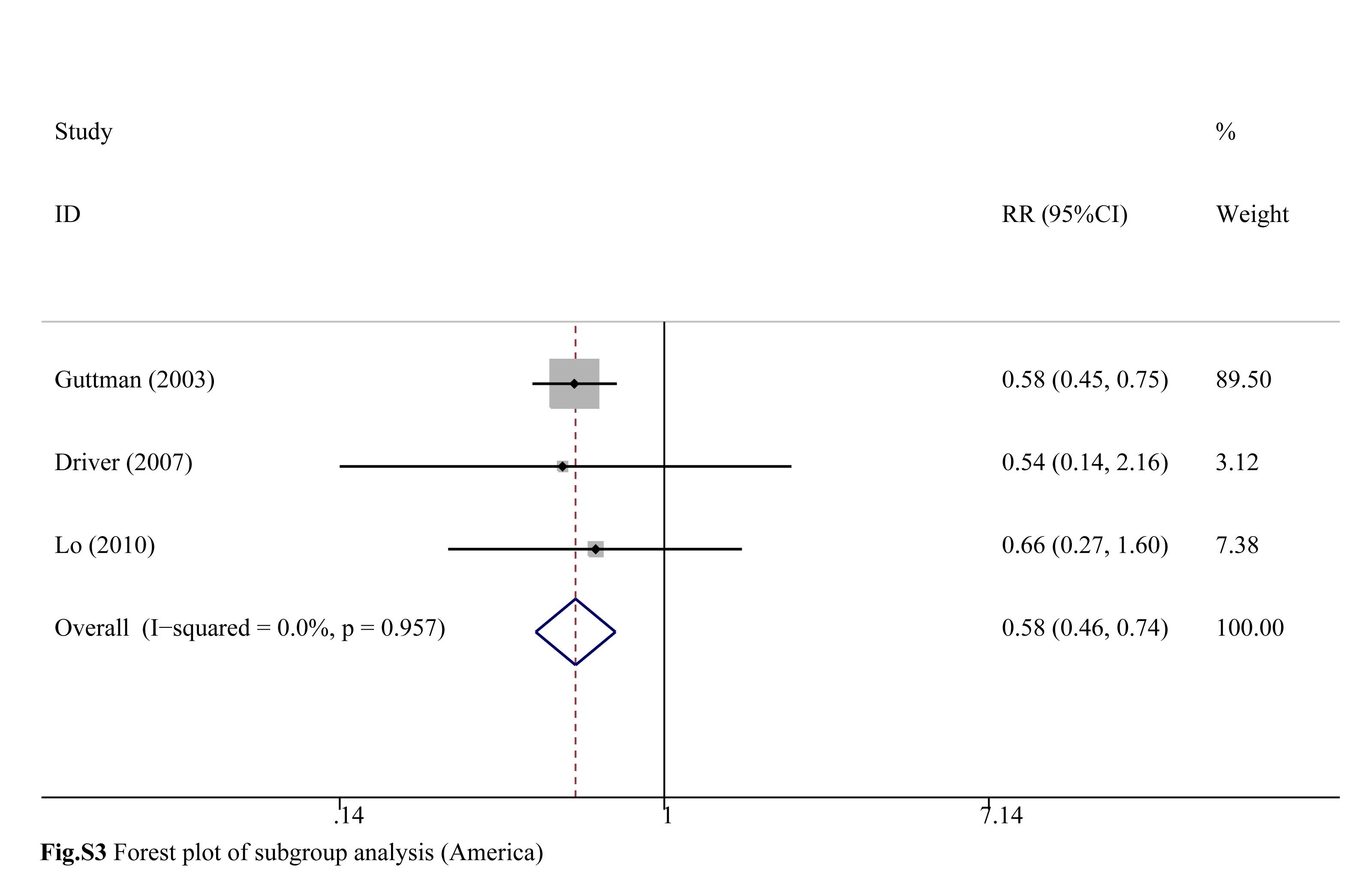


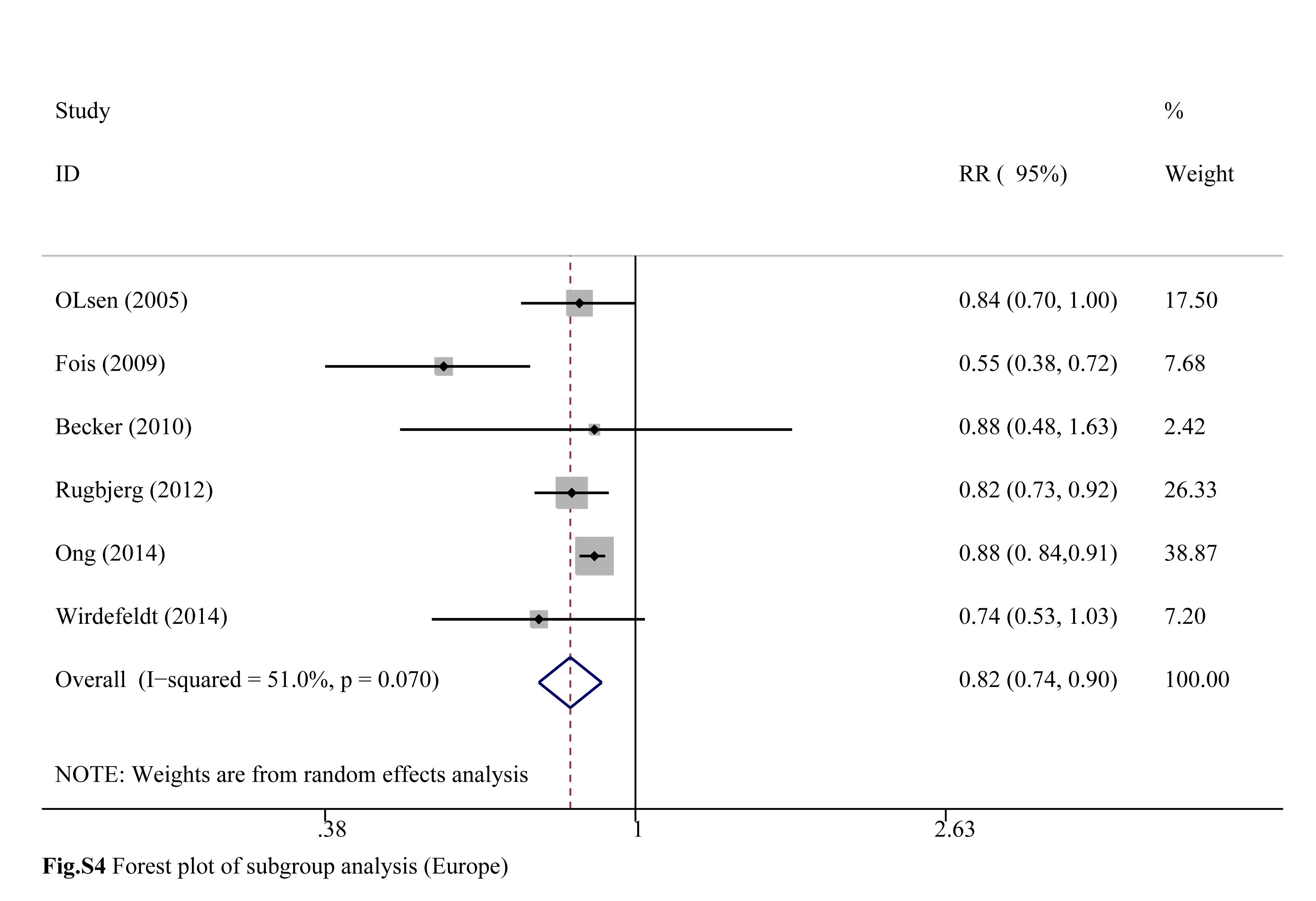


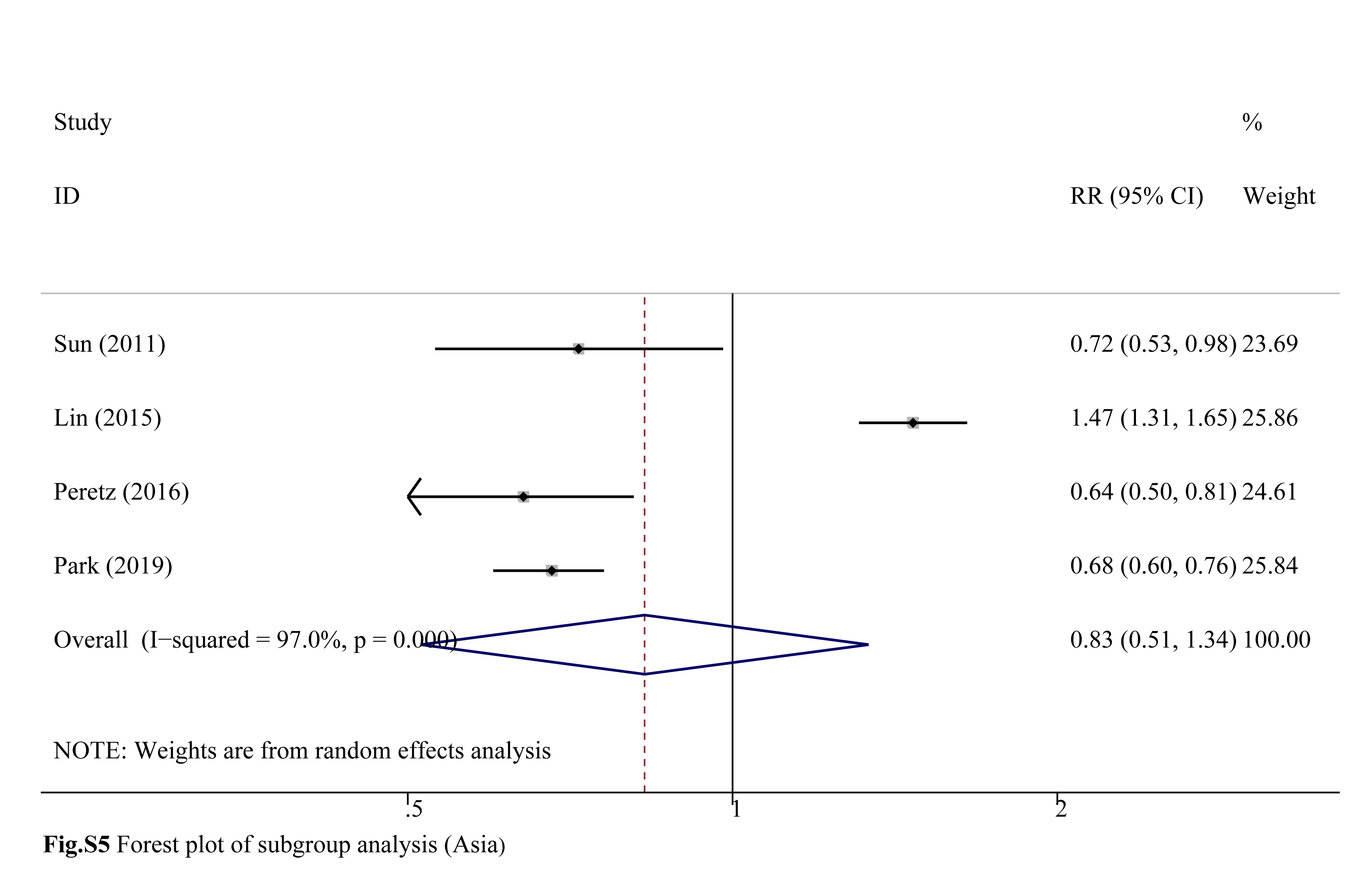


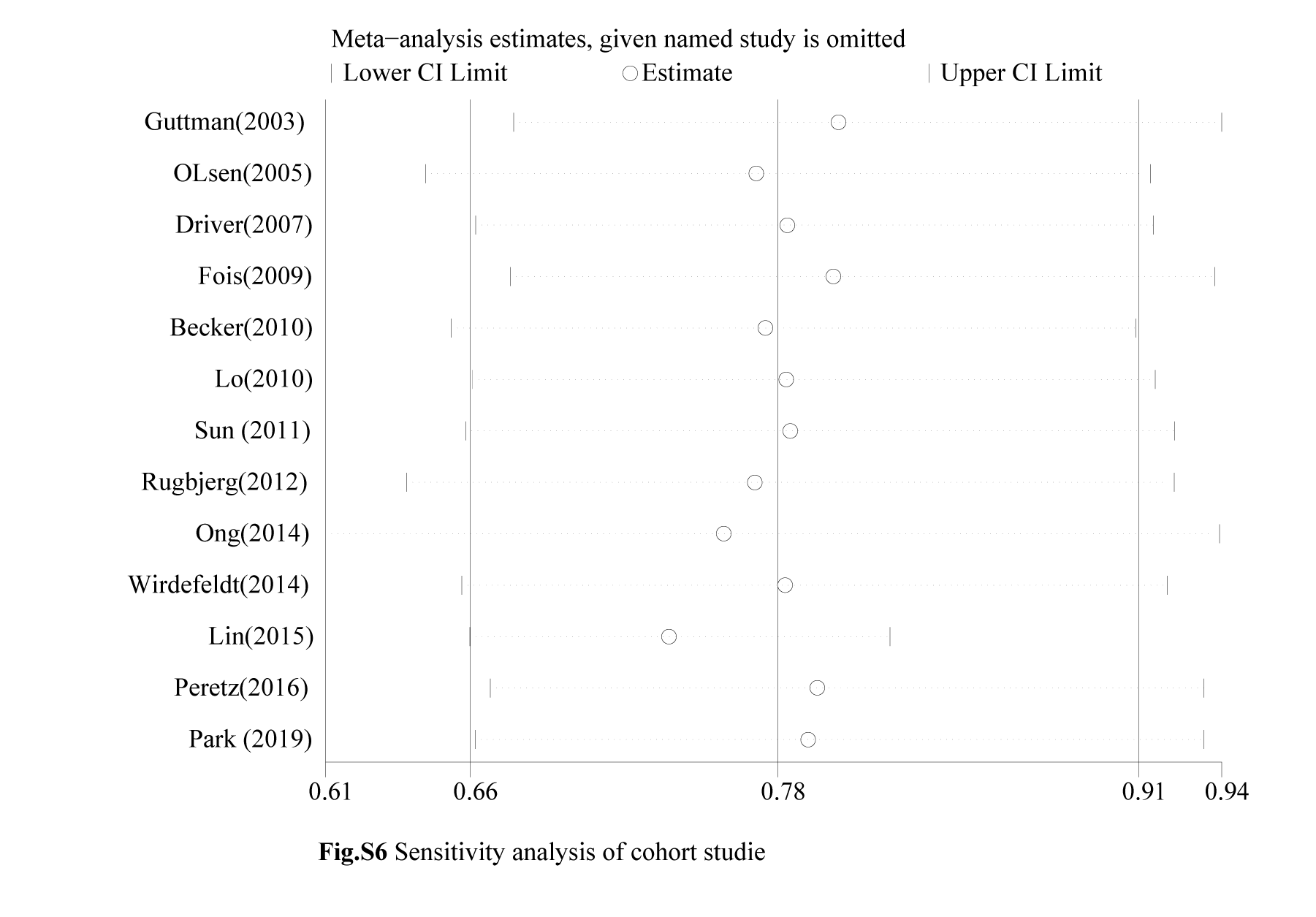


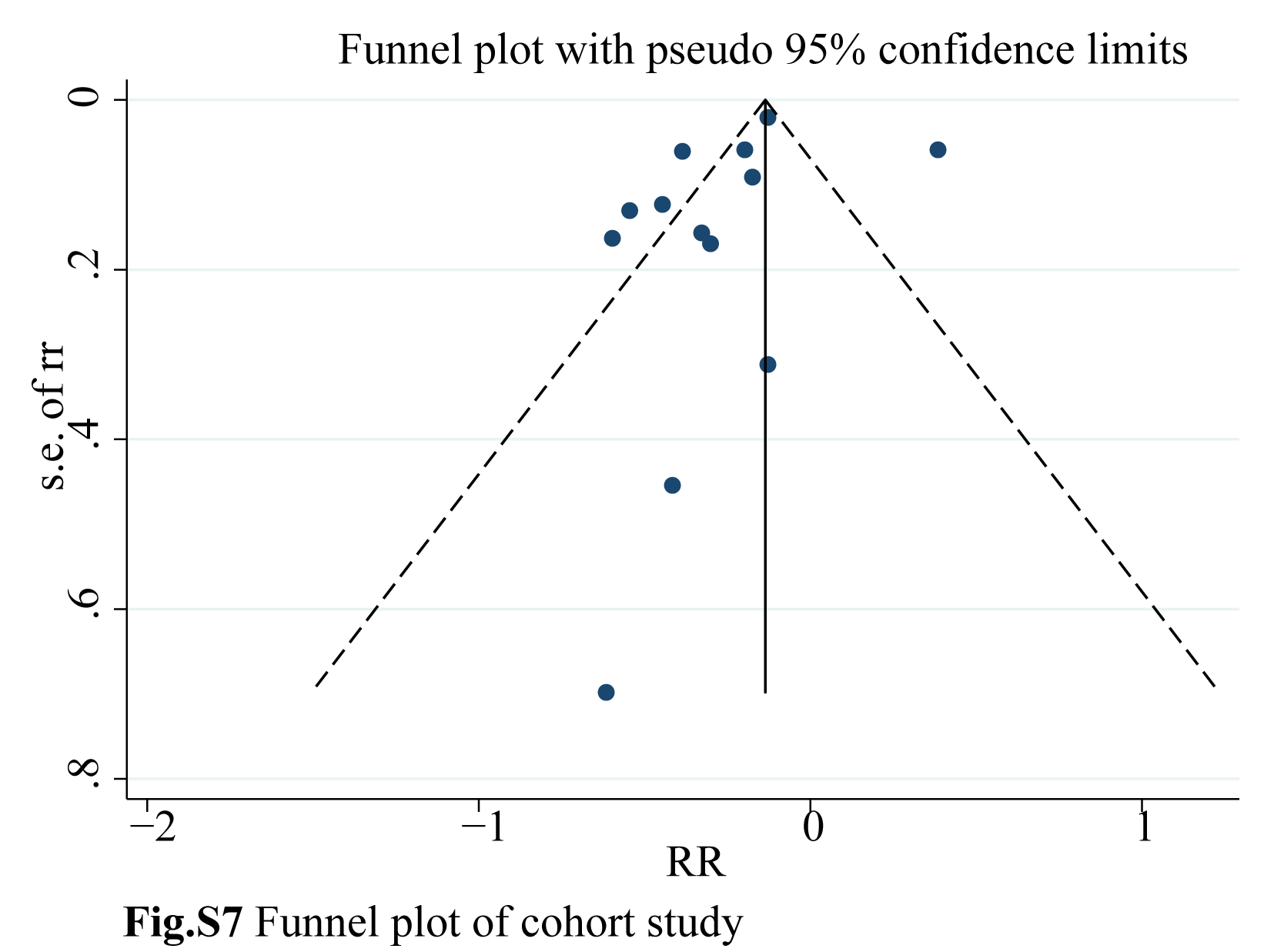


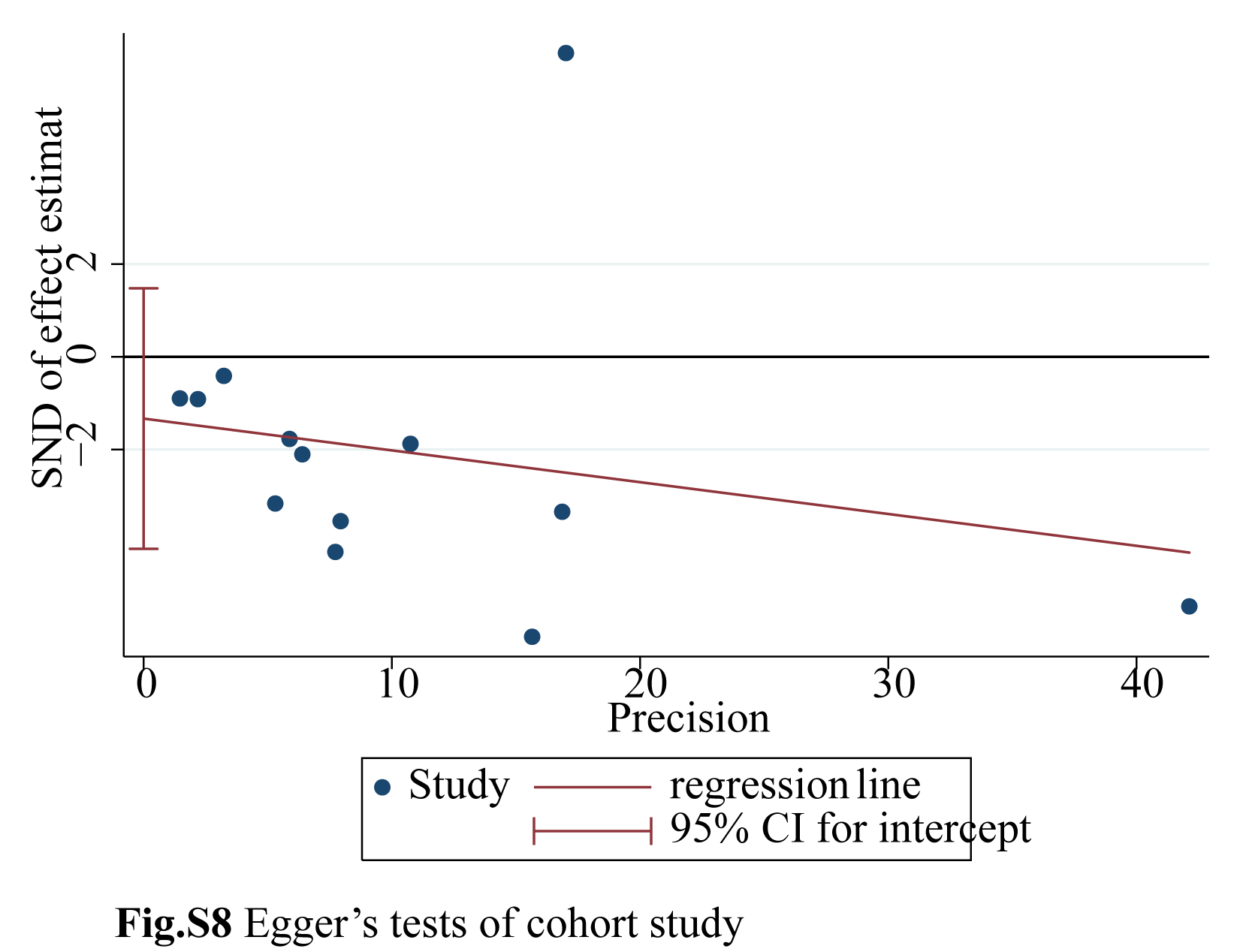

Supplement: Supplementary file 1 — Additional file 1: Fig. S1 Forest plot of subgroup analysis (colon). Fig. S2 Forest plot of subgroup analysis (rectum). Fig. S3 Forest plot of subgroup analysis (America). Fig. S4 Forest plot of subgroup analysis (Europe). Fig. S5 Forest plot of subgroup analysis (Asia). Fig. S6 Sensitivity analysis of cohort study. Fig. S7 Funnel plot of cohort study. Fig. S8 Egger’s tests of cohort study. [file 12877_2021_2497_MOESM1_ESM.docx]
