# Supplementary material for: Patients with Parkinson’s disease predict a lower incidence of colorectal cancer
Source: BMC Geriatr. 2021 Oct 18;21:564. doi: 10.1186/s12877-021-02497-z (PMC8522030; doi:10.1186/s12877-021-02497-z)
Supplement: Supplementary file 2 — Additional file 2: Table S1 The quality of the included studies assessed by NOS. [file 12877_2021_2497_MOESM2_ESM.docx]

**Patients with** **Parkinson’s disease predict a lower incidence of colorectal cancer**

Hongsheng Fang, Yunlan Du, Shuting Pan, Ming Zhong, Jiayin Tang

**Table S1. The quality of the included studies assessed by NOS**

| Study/year | Selection | | | | Control for important factors* | Exposure | | | NOS Score |
| --- | --- | --- | --- | --- | --- | --- | --- | --- | --- |
|  | Adequate definition of cases | Representativeness of cases | Selection of controls | Definition of controls |  | Ascertainment of exposure | Same method to ascertain for cases and controls | Nonresponse rate |  |
| Guttman 2003 | 1 | 0 | 1 | 1 | 1 | 1 | 1 | 0 | 6 |
| OLsen 2005 | 1 | 0 | 1 | 1 | 1 | 1 | 1 | 0 | 6 |
| Powers 2005 | 1 | 1 | 0 | 1 | 1 | 1 | 1 | 0 | 6 |
| Driver 2007 | 1 | 1 | 1 | 1 | 1 | 0 | 1 | 0 | 6 |
| Fois 2009 | 1 | 0 | 1 | 1 | 2 | 1 | 1 | 0 | 7 |
| Becker 2010 | 1 | 1 | 1 | 1 | 1 | 0 | 1 | 0 | 6 |
| Lo 2010 | 1 | 1 | 1 | 1 | 2 | 1 | 0 | 0 | 7 |
| Sun 2011 | 1 | 1 | 0 | 1 | 1 | 1 | 1 | 0 | 6 |
| Rugbjerg 2012 | 1 | 0 | 1 | 1 | 1 | 1 | 1 | 1 | 7 |
| Ong 2014 | 1 | 1 | 1 | 1 | 1 | 1 | 1 | 1 | 8 |
| Wirdefeldt 2014 | 1 | 1 | 1 | 1 | 1 | 1 | 1 | 0 | 7 |
| Lin 2015 | 1 | 1 | 1 | 1 | 1 | 1 | 1 | 1 | 8 |
| Peretz 2016 | 1 | 1 | 1 | 0 | 1 | 1 | 1 | 1 | 7 |
| Boursi 2016 | 1 | 0 | 1 | 1 | 1 | 1 | 1 | 0 | 6 |
| Freedman(1) 2016 | 1 | 1 | 0 | 0 | 1 | 1 | 1 | 0 | 5 |
| Freedman(2) 2016 | 1 | 1 | 0 | 0 | 1 | 1 | 1 | 0 | 5 |
| Park 2019 | 1 | 1 | 1 | 1 | 1 | 1 | 1 | 1 | 8 |

**A maximum of two stars can be allotted in this category, one for Age, sex, the other for other controlled factors.*
